# Plant-Derived Natural Product-Based Nanoformulations for Healthcare Application

**DOI:** 10.7150/ntno.113606

**Published:** 2025-08-16

**Authors:** Saloni Kakkar, Rajan K Jha, Deeksha Hattila, Abhishek Kumar Singh, Pradeep K Shukla, Shiv Pratap Singh Yadav, Avtar S. Meena

**Affiliations:** 1 CSIR-Centre for Cellular and Molecular Biology (CCMB), Uppal Road, Hyderabad, Telangana, India.; 2 Department of Physiology, College of Medicine, University of Tennessee Health Science Center, Memphis, TN, USA.; 3 Manipal Center for Biotherapeutics Research, Manipal Academy of Higher Education, Karnataka, Manipal, 576 104, India.; 4 Department of Biotechnology, All India Institute of Medical Sciences (AIIMS), New Delhi, India.

**Keywords:** Natural products, Nanotheranostics, Healthcare, Nanoparticles, Diabetes, Cancer, Immunomodulation.

## Abstract

Plants produce numerous natural compounds developed into commercial products. These compounds offer medicinal benefits for treating diseases like diabetes, neurological disorders, malaria, and cancer. They also serve as hepatoprotective agents and immunomodulators. These natural products are secondary metabolites that plants produce for their defense and adaptation. Recently, numerous reports have highlighted the effectiveness of natural products in different diseases. However, comprehensive analysis of plant-based products currently used in the clinical setting for various human diseases is insufficient. This review provides extensive information about the application of natural plant products in both clinical and preclinical settings. It highlights their role in developing drugs for human diseases. Additionally, these plant products could serve as diagnostic tools for various diseases. Plant-derived natural products, integrated with advanced nanotechnology-based approaches, could enhance healthcare monitoring without compromising treatment efficacy. Nanotechnology techniques employing both diagnostics and therapeutics, known as nanotheranostics, utilize engineered biocompatible nanomaterials with potential prospects for healthcare management. Nanomaterials like polymeric nanoparticles and liposomes offer diagnostic value by enabling real-time imaging of disease progression and treatment response. Functionalized with contrast agents or dyes, they enhance MRI, CT, PET, and fluorescence imaging, improving diagnosis, patient stratification, and monitoring of drug delivery and efficacy. With the increasing demand for natural dietary supplements, this issue encompasses the identification of various plant-based natural products as potential nanotheranostics with promising potential for chronic disorders such as cancer, neurological pathologies, diabetes, and immunological issues. This review focuses on applications of nanotheranostics utilizing natural products in biomedical applications, outlining the current breakthroughs, supplemented with future potentialities.

## Introduction

**Figure 1:**
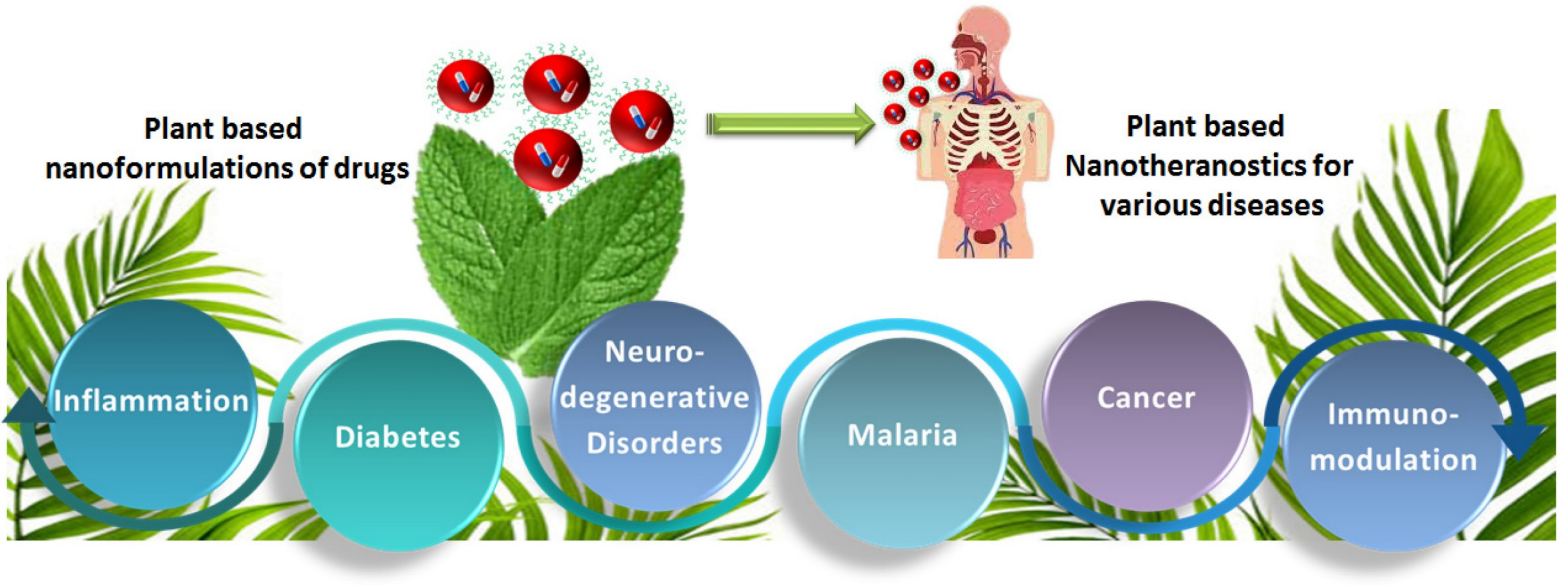
Schematic illustration displaying various diseases for which plant-based natural products and their nanotheranostics are discussed in the current report.

**Figure 2:**
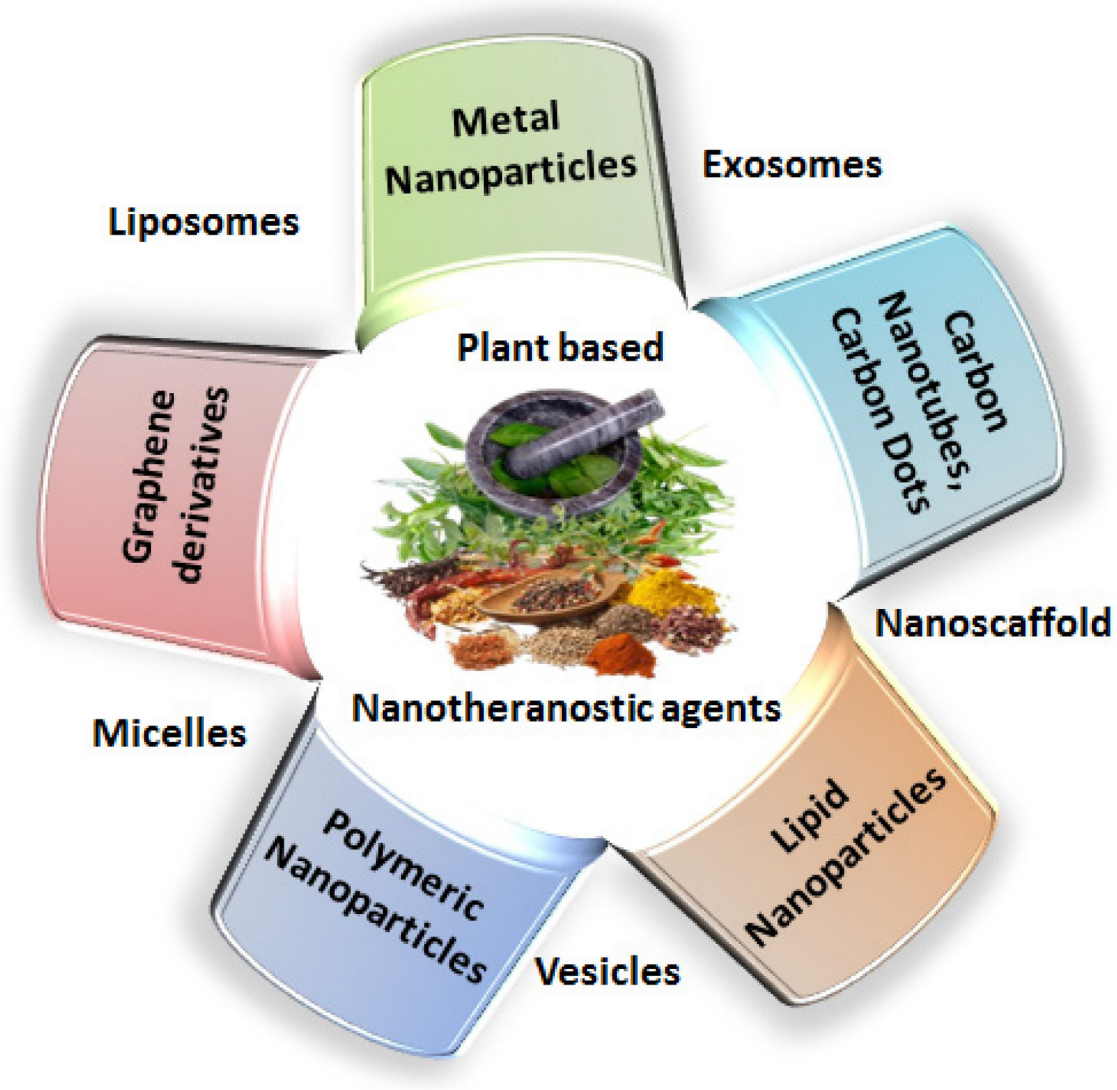
Various nanotheranostic agents, such as nanomaterials as metallic and polymeric nanoparticles and other biological nanosystems, that are utilized to design plant-based nanotheranostics for healthcare applications.

**Figure 3:**
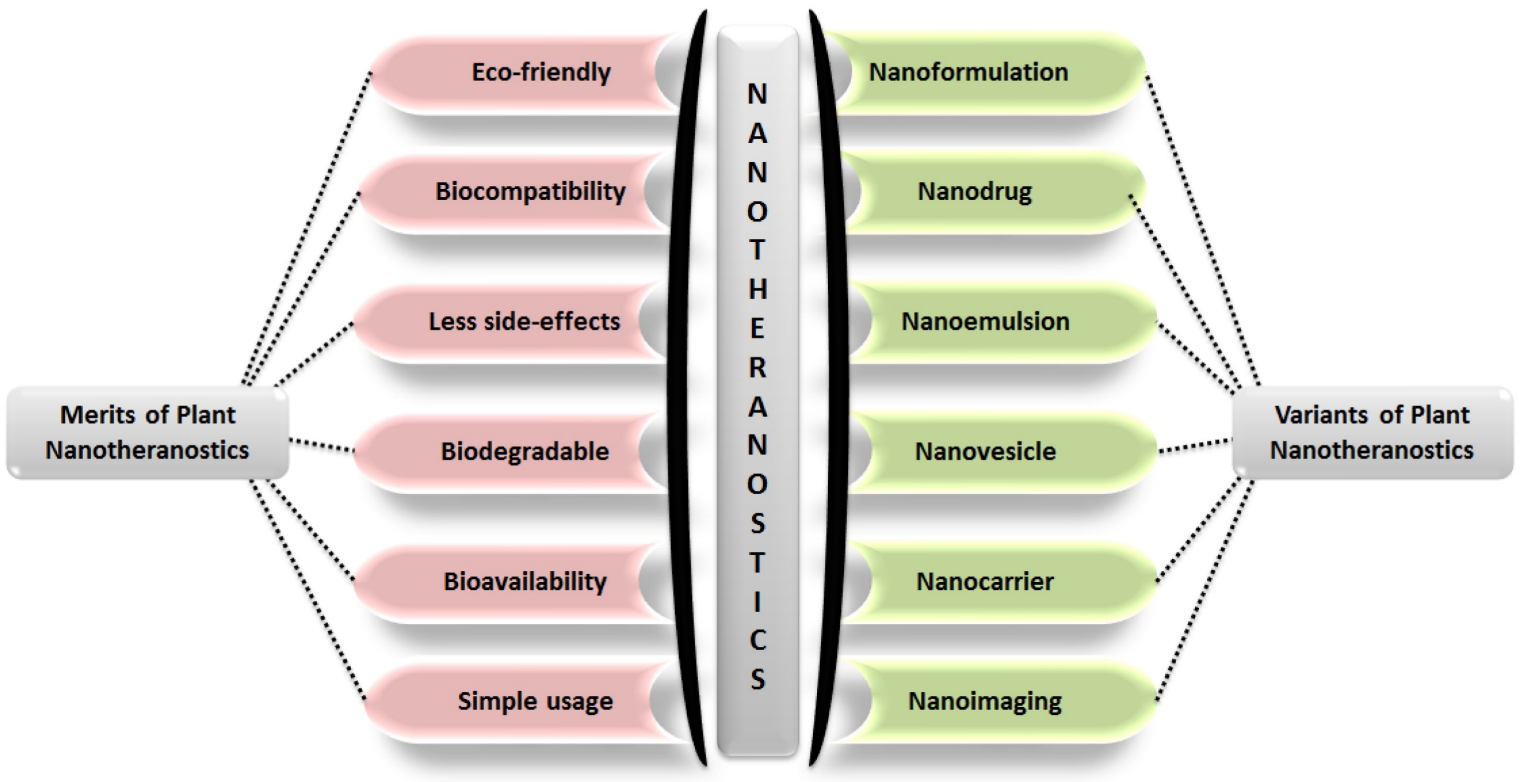
A diagrammatic compilation of different advantages offered by plant product-based nanotheranostics (left) along with various nanotheranostic variants that are designed from plants and their natural products.

India is home to over 45,000 plant species, with around 20,000 recognized for their medicinal potential. Of these, approximately 7,500 are used by traditional communities in healthcare practices, representing the highest percentage of medicinal plant use globally 1, 2. Ayurveda, India's ancient medical system, identifies around 2,000 such plants, while Siddha and Unani recognize 1,121 and 751, respectively. Roughly 25% of all drugs are plant-derived 3. In contrast, modern western medicine remains inaccessible and unaffordable for many worldwide. Despite over a century of western medical influence in India, traditional medicine continues to serve as the primary healthcare resource for a large population. The World Health Organization (WHO) supports the use of indigenous systems based on locally available medicinal plants. Notably, about 50% of drugs in the United States contain one or more natural products 4. Natural compounds are generally classified into primary and secondary metabolites. Primary metabolites—such as nucleic acids, amino acids, sugars, and fatty acids—are essential for basic life processes. Secondary metabolites, on the other hand, are bioactive compounds that support ecological functions and often provide therapeutic benefits. These are extracted from diverse biological sources including plants, marine organisms, and microorganisms. Within raw extracts, novel bioactive molecules—referred to as active ingredients—can be identified. Many plant-derived secondary metabolites demonstrate therapeutic benefits for a range of diseases. To enhance their effectiveness and overcome delivery limitations, researchers are increasingly applying nanotechnology-based solutions. Nanotheranostics refers as integrated nanotechnology platform that combines diagnostic and therapeutic functionalities within a single system, enabling simultaneous imaging, targeted drug delivery, and real-time monitoring of treatment efficacy 5. These multifunctional systems allow for the targeted delivery of both diagnostic agents and therapeutics, offering a significant advantage over traditional methods. They have shown promise in managing various acute and chronic conditions, including cancer, diabetes, neurological disorders, and immune-related diseases 6. To address these challenges, plant-derived nanomaterials—such as nanoparticles, lipid-based carriers, magnetic conjugates, silica encapsulates, and carbon-based structures—act as effective nanotheranostic agents. These nanotechnology-fused plant-based nanomedicines, nanodrugs, nanophenols, the green synthesis of nanoparticles, etc., display improved potential for detecting and targeting specific body tissues/cells with promising therapeutic potential 7. Nanotechnology plays a crucial role in boosting the performance of herbal medicines, especially those with poor solubility, low intestinal permeability, rapid metabolic breakdown, or limited systemic absorption. Nanoformulations help address these issues, ensuring more effective delivery to target sites 8. These nanoforms can also be utilized for diagnosis along with treating disease. This involves delivering diagnostic agents to specific targets and subsequently visualizing their distribution by imaging techniques 9-11. Nanoparticles' large surface area enables them to carry multiple targeting ligands, imaging probes, and therapeutic agents, providing a flexible platform for noninvasive diagnostics and enhancing diagnostic accuracy through various imaging modalities 12. Chemical and biological markers, when linked to nanoparticles, offer a flexible platform for noninvasive diagnostics. These markers provide both qualitative and quantitative data through imaging modalities such as Computed Tomography (CT), Magnetic Resonance Imaging (MRI), Positron Emission Tomography (PET), and Near-infrared (NIR). These imaging techniques can respond to variety of stimulus, such as magnetic fields, temperature, ultrasound, electric pulses, and internal factors such as glucose levels, pH, and redox conditions, increasing diagnostic accuracy and patient comfort 13. The draft uniquely consolidates recent advancements in the design and application of plant-derived natural product-based nanoformulations across diverse healthcare domains, as represented in **Fig. 1**. A diverse range of nanotheranostic agents, have been applied to develop natural product-based nanotheranostics for healthcare 14. In addition, reports and studies related to the use of plant-based exosomes, micelles, nanovesicles, nanoscaffolds, etc., as nanotheranostic agents for biomedical disorders are elaborately discussed and illustrated in **Fig. 2**. This review also discusses the different nanotheranostic variants of plants and their secondary metabolites, which offer a wide range of advantages over conventional and synthetic nanotechnology for biomedical management, as graphically compiled in **Fig. 3**. Unlike existing literature that often focuses on individual classes of phytoconstituents or specific diseases, our review offers a comprehensive perspective spanning phytochemical diversity, nanocarrier systems, and translational relevance. By integrating insights into formulation strategies, therapeutic mechanisms, and clinical promise, this review aims to bridge the gap between phytomedicine and precision nanotheranostics.

### Natural product-based nanotheranostics for treating diabetes

Diabetes is a significant health problem that is associated with a high risk of various complications. Hyperglycemia is characterized by elevated blood glucose levels. Persistent hyperglycemic illness leads to neuropathy, stroke, nephropathy, and cardiovascular disease (CVD) in the context of Type 2 Diabetes Mellitus (T2DM). As a result, controlling blood glucose levels is a crucial approach for the prevention of T2DM. Herbs, beans, and onions contain polyphenols, which improve digestive issues, neurodegenerative disease, cardiovascular complications, and diabetes 15. One report suggested that onion has anti-inflammatory, antioxidant, and antidiabetic effects because of the presence of flavonoid like quercetin and organosulfur compounds 16-18. The Amadori rearrangement compound obtained from heat-processed onion extract suppressed the absorption of carbohydrates by blocking intestinal sucrose, thereby reducing blood glucose levels 19. Cannabinoid receptor 1 (CB1R) activation exacerbates insulin resistance and hyperglycemia by inducing endoplasmic reticulum stress and gluconeogenesis. Gomisin, a compound from Schisandra chinensis, ameliorates CB1R-mediated insulin dysfunction and glucose metabolism abnormalities *in vitro*. *In vivo* studies demonstrated that gomisin attenuates hyperglycemia induced by a high-fat diet, insulin resistance, and endoplasmic reticulum (ER) stress by inhibiting CB1R signaling and ceramide biosynthesis 20. A few examples of plant products that exhibit antidiabetic properties are discussed in Table 1 below. The field of nanotheranostic strategies for diabetes management is rapidly advancing and holds the potential to transform diabetes management. By combining diagnostics and therapeutics with a single nanoparticle, this approach enables targeted drug delivery, improved glucose monitoring, and enhanced wound healing. Nanotheranostics offer promising prospects for improving the lives of people with diabetes 21. Various studies have reported polymeric nanoparticle-based delivery of insulin via oral and nasal routes. This affects treatment physiology via age-related metabolic variations that in turn influence the bioavailability and release of insulin 22. One dietary bioflavonoid, quercetin, has been studied as a superparamagnetic iron oxide nanoparticle conjugated with quercetin (QCSPIONs) for reducing blood glucose levels in diabetic rat models. The authors demonstrated that the nanoconjugate-driven regulation of microRNA-29, which elevates the expression of glucose transporters and insulin-like growth factor-1, plays a crucial role in preventing diabetic complications 23, 24. Cheng et al. (2020) reported a green synthesis method for producing gold nanoparticles using an extract from *Ramulus mori* plant leaves and methanol. The plant extract was conjugated onto polyacrylic gold nanoparticles, which were developed as a novel drug delivery system for gestational diabetes. A large variety of nanoformulations have been fabricated for diabetes, ensuring efficient targeted drug delivery with the required release system. Additionally, targeted ligand-based delivery via nanocarriers increases drug availability and stability, thus reducing the dosage and administration frequency 25.

### Natural product-based nanotheranostics for treating neurodegenerative diseases

Neurodegenerative diseases are significant issues impacting millions of people globally 26. These diseases are marked by the degeneration of neurons in the central nervous system, affecting particular brain functions. The most notable neurodegenerative disorders include Huntington's disease (HD), Parkinson's disease (PD), amyotrophic lateral sclerosis (ALS), Alzheimer's disease (AD), multiple sclerosis (MS). The origin and pathophysiology of these neurodegenerative diseases are unspecified; however, it is widely believed that these disorders have common underlying causes at the cellular and molecular level, including: oxidative stress, chronic inflammation, mitochondrial dysfunction, disrupted calcium balance, and protein misfolding 27-29.

Unfortunately, in this context, there are no therapeutic interventions available to halt the progression of these diseases. However, various studies have shown that natural products can be beneficial in treating neurodegeneration, which may be promising candidates for potential therapies. For example, extracts isolated from *Lippia citriodora* (VEE) and verbascoside (Vs), which are phenylpropanoid glycosides, exert relaxation effects by regulating genes responsible for maintaining calcium homeostasis and energy production 30. An extract isolated from *Chionanthus retusus* blocked nitric oxide (NO) production induced by lipopolysaccharide (LPS) and showed neuroprotective effects against glutamate-induced cellular toxicity 31. Moreover, auraptene (AUR), a 7-geranyloxylated coumarin isolated from citrus fruit, mitigated PD symptoms by inhibiting mitochondrial respiration and reducing reactive oxygen species (ROS) generation 32. Table 2 comprises studies describing various plants that are effective for treating neurodegenerative disorders. Nanotechnology solutions for brain-related disorders employ specially designed materials and devices that operate within biological systems at the molecular level. Nanoconjugates derived from medicinal plants and their extracts have played a crucial role in the advancement of innovative neurodiagnostic and therapeutic approaches, offering potential alternatives to plant-based nanotheranostics. Their advantages include their natural abundance, targeted delivery of specific molecules to the brain, and enhanced efficacy with a decreased likelihood of side effects 33. The efficacy and pharmacokinetic properties of plant products combined with nanoparticle-based delivery systems are enhanced. These systems can traverse the blood-brain barrier (BBB) as nanotheranostic agents for PD have been shown to be enhanced 34. Marine algae have been extensively investigated for their wealth of bioactive nutraceuticals. Various biological and environmental factors influence the production of primary and secondary metabolites. It serves as an efficient biofactory for various theranostic compounds, and its alignment with nanomaterials is easy to handle, has the capacity to conjugate inorganic metallic ions, has a lower cost and, most importantly, is an eco-friendly, healthier synthesis of NPs. Marine algae-based nanotheranostics are excellent stabilizing agents for the green synthesis of thermodynamically stable nanoparticles 35. Another study related to nanoparticles containing curcumin, piperine along with glyceryl monooleate (GMO) demonstrated effective penetration and sustained release into brain tissue across the BBB, indicating an anti-Parkinsonism effect. A previous study in PD model mice revealed that rotenone impaired coordination of motor neurons and controlled dopaminergic neuronal degeneration 36. They also prevent the aggregation of α-synuclein, death of dopaminergic neurons and mitochondrial dysfunction 34. Additionally, it has been found that lipid nanoparticles can improve the absorption and utilization of plant-based nutraceuticals, owing to their ease of administration via oral, nasal, parenteral and topical routes 37. Many such reports are described in Table 3 below, employing nanotheranostic agents for neurodegenerative disorders.

### Natural product-based nanotheranostics for Malaria

Malaria is a life-threatening and endemic disease transmitted by the bite of the *Anopheles* mosquito 38. It is more prevalent in tropical countries such as Asia, Africa, and Latin America. Malaria remains a global health challenge, affecting nearly half the world's population. The World Health Organization estimated that 229 million people contracted malaria in 2019, resulting in 409,000 deaths. Plasmodium parasites cause this disease, while *Plasmodium falciparum* being the deadliest strain 39*.* Malaria disproportionately affects young African children and hinders economic growth. Drug resistance complicates treatment and control efforts 40. Hence, there is an urgent need for novel and herbal antimalarial products obtained from medicinal plants. The first compound in this category is quinine, which was isolated from *Cinchona spp*. and is still used as an effective antimalarial drug. Studies suggest that quinine, interfere with hemoglobin digestion during the blood stage of malaria infection. Despite the resistance of *P. falciparum* to quinine derivatives, quinine is routinely used to manage Plasmodium infection 41. The second herbal antimalarial drug, artemisinin (ARS), was first derived from the traditional Chinese medicine *Artemisia annua* 42. The structural backbone comprises a sesquiterpene lactone having a peroxide bridge that possesses excellent antimalarial activity and is also active against quinine-resistant *P. falciparum* 43, 44. Furthermore, Artemisinin-based Combination Therapies (ACTs) are reported to provide better efficacy in the case of multidrug-resistant malaria 44, 45. Subsequently, several alkaloid compounds isolated from various medicinal plants were reported to have antimalarial activity. These alkaloids possess profound structural heterogeneity and include indoles, aporphines, steroids, terpenoids, phenanthroindolizidines, hasubanane, isoquinoline, benzylisoquinoline, naphthoisoquinoline, morphinandienone, protoberberine, amaryllidaceae, cyclopeptides, quinolines, pyridocoumarins, acridones, and macrocyclic alkaloids 40. An Indian study described sixty-eight plant species belonging to 33 families used to eradicate malarial infection in northeast India 46. These plants either kill the parasite directly or act as a hepatic shield when combined with other plant species. Some commonly used antimalarial plant species in northeast India include *Coptis teeta, Ocimum sanctum, Vitex peduncularis, Alstonia scholaris, Crotalaria occulta, and Polygala persicariaefolia.* In addition, few plant species, such as* Eucalyptus globule, Ocimum gratissimum, Homalomena aromatica, and Elsholtzia blanda,* are described as mosquito repellents. However, whether these plant species act as repellents, insecticides or both has yet to be determined. Furthermore, several studies highlighted the effectiveness of plant-based repellents in preventing malaria 47, 48. Plant-based medicines have exhibited promising effectiveness when combined with nanotechnology-based delivery systems, leading to better management of malarial infections 49. Various plant parts, like leaves, roots, latex, seeds, and secondary metabolites, have been used as eco-friendly candidates for the green synthesis of nanoparticles. Plants and their natural products act as sources of reducing molecules for bio-nanosynthesis, contributing to cost-effectiveness and reduction of any harmful chemical wastes. The green-synthesis of Ag-nanoparticles using *Bruguiera cylindrical, Centroceras clavulatum,* and* Moringa oleifera* has been reported to have antimalarial and antidengue effects 50. *Ramanibai et. al*., conducted a study on the larvicidal efficacy of silver nanoparticles synthesized from 2,7-bis[2-[diethylamino]-ethoxy] fluorene, isolated from *Melia azedarach* leaves, against *Culex quinquefasciatus* and *Aedes aegypti*. This method was adopted as a novel alternative that can be employed for mosquito control 51. Suman *et. al.,* explored how well titanium dioxide nanoparticles, derived from *Morinda citrifolia* root extract, could eliminate mosquito larvae. They tested these nanoparticles on three mosquito species—*Anopheles stephensi, Aedes aegypti,* and *Culex quinquefasciatus*—while also evaluating their safety for non-target fish 52. Table 4 lists various herbal plants and their components employed to design nanotheranostic platforms for controlling malaria and parasitic infections.

### Natural product-based nanotheranostics for cancer

Cancer is a complicated disease marked by the abnormal and uncontrolled growth and spread of cells. As a major global health issue, it is the 2^nd^ most common cause of death worldwide, surpassed only by CVD. With over 100 identified types, cancers are classified on the basis of their origin. Lung, prostrate and colorectal cancers are the most frequently occurring cancers among men, whereas breast, lung, and colorectal cancers are the leading types in women 53. In 2020, the total number of cancer cases was 19.3 million, accounting for 10.0 million deaths globally (GLOBOCAN 2020 database). Furthermore, the burden of cancer is increasing worldwide because of poor diagnosis and management. This global surge in cancer cases has substantially affected the country's economic status and individual physical and emotional well-being. Hence, the cancer burden can be reduced by early diagnosis and appropriate management of the patient. One approach for cancer management is chemoprevention, which involves the use of synthetic, natural molecules (such as plant extracts) and biological agents to prevent carcinogenesis at one of the steps of tumor progression: initiation, promotion, and progression 54, 55. Medicinal plants harbor a mixture of bioactive compounds with potent anticancer activity and have been used in cancer treatment for centuries 56, 57. The daily consumption of coffee is very high worldwide. The chemoprotective role of coffee has been established for a variety of cancers. This role can be imparted by various compounds, such as caffeine, diterpenes, and chlorogenic acid, present in coffee 54. Since coffee is a mixture of several compounds, the exact molecular mechanism of individual compounds and other confounding factors is yet to be determined. High intake of dietary carotenoids, including β-carotene, curcumin, lutein, phytoene, crocin, crocetin, lycopene, β-cryptoxanthin, and astaxanthin, has been reported to reduce the risk of breast, ovarian, cervical, and colorectal cancers 58, 59. Different carotenoids tend to target different pathways to regulate cancer progression. The molecular mechanisms underlying the chemoprotective function of carotenoids include apoptotic induction, targeting of gap junction intercellular crosstalk, regulating cell cycle progression, exerting antiproliferative effects, reducing the number of Bcl-2 and Bcl-xl positive cells, modulating growth factor signaling, antioxidant response elements and regulating the expression of differentiation-related proteins 58. Resveratrol is a natural nonflavonoid polyphenol present in grape skin, peanuts, *Polygonum cuspidatum,* and other plants and fruits. Resveratrol possesses robust antitumor activity and has been implicated in managing various cancers, including skin, liver, breast, colon cancer 60, 61. In a recent study, resveratrol, when used as a combination therapy with 5-FU (fluorouracil) against colon cancer cell lines (SW480 and LoVo), was shown to reduce drug resistance by modulating apoptosis through the BAX gene 61. Resveratrol was found to suppress the metastasis of human gastric cancer cells by inhibiting the metastasis-associated lung adenocarcinoma transcript 1 (MALAT1)-mediated epithelial-mesenchymal transition (EMT) in the BGC823 cell line 62. Green tea is a rich source of polyphenols, particularly epigallocatechin-3-gallate (EGCG). The active biomolecule EGCG has been implicated in various cancers, including prostate, brain, bladder, and cervical cancers 63, 64. Experimental evidence from human hepatoma cell lines (HepG2 and Huh7) and hepatocellular carcinoma (HCC) rat models treated with EGCG suggested that EGCG inhibited cell growth and prolonged the lifespan of rats. by increasing the levels of p21waf1/Cip1 and downregulating CDC25A levels 65 Furthermore, several case‒control, epidemiological, and meta-analyses have been shown to reduce the risk of prostate cancer in a cohort with high tea intake 66. Accumulating evidence from *in vitro* and *in vivo* experiments also suggests that EGCG can induce cancer cell apoptosis via epigenetic regulation of apoptosis-associated genes such as human telomerase reverse transcriptase (hTERT) or by increasing the levels of ROS 67. A wide range of natural products with anticancer properties are listed in **Table 5.** The field of cancer nanotheranostics has utilized nanoparticles because of their capacity to accumulate at tumor locations as substitutes for conventional chemotherapy approaches and medical treatments. Compared with chemotherapy, natural compounds seem to offer a choice with fewer adverse effects; however, concerns about their bioavailability persist. NPs combined with medicinal and herbal plants are used as drug and gene transporters to tumor sites, as imaging agents offering reduced toxicity and effective biocompatibility. The small size of plant nanoconjugates can easily negate side effects after their administration into tumor tissues, prolonging their sustained release and enhancing their effectiveness. Moreover, coupling natural product nanoconjugates with specific receptor moieties can result in active targeting of tumor tissues 68. *Chelora* et al*.* studied the chemotherapeutic effect of piperine extract from pepper (Pip) via self-assembly and the formation of nanoparticles with PEG (Pip NPs). Pip NPs exhibit excellent cancer-killing properties and have emerged as cost-effective anticancer herbal nanotheranostics. Carbon dots (CDs), a major group of nanotheranostic agents, have been extensively reported for their green synthesis via various plant-based materials, such as garlic, fruit extract, mulberry, oils, and *Buchanania lanzan*, as substrate precursors 69. The medicinal plant-based synthesis of CDs is a one-pot, cost-effective synthesis with highly tunable properties, and its biocompatibility has attracted the attention of many researchers 70. Fresh ginger juice has also been reported to suppress the growth of human hepatocellular carcinoma cells (HepG2) via the production of reactive oxygen 71, 72 Carbon nanotubes (CNTs), another class of nanotheranostic agent, can be produced using diverse natural hydrocarbon precursors, such as plant extracts (including tea-tree extracts), essential oils (including sunflower oils, eucalyptus oil), milk, honey, biodiesel, eggs, and other materials 73. Other examples of such plant-based anticancer nanotheranostics are listed in Table 6.

### Natural product-based nanotheranostics for immunomodulation

The immune system plays a vital role in protecting individuals from diseases and toxins. An altered or compromised immune function is associated with several conditions. Immunomodulators possess the ability to modulate immune function. Immunomodulators can be synthetic (such as chemical compounds) or natural (such as plant products) 3. Plant-based natural immunomodulators have been explored extensively in the past few decades. Interestingly, their use is rooted in traditional medical systems and is now implicated in various pathological conditions. Herbal immunomodulators can exert their effects by directly acting on the pathogen or indirectly by modulating the immune system, *i.e.*, both native and acquired immunity, of the host. Broadly, immunomodulators can be categorized into three major classes: a) immunostimulatory agents, b) immunosuppressive agents, and c) tolerogens 3. Immunostimulatory molecules are used in cases of immunodeficiency, infections, and cancers and act by stimulating or augmenting the molecules of the immune system. In contrast, immunosuppressive agents are used in autoimmune diseases or transplantation and work by weakening the immune system. Tolerogens tend to confer immunological tolerance and make the immune component nonresponsive to the antigen. The immunostimulatory class of immunomodulators is more common than the other two types 74. Although the precise molecular mechanism of herbal immunomodulators is not well understood, they are known to regulate pro-inflammatory and anti-inflammatory cytokines. Another mechanism, such as modulation of the gut microbiome, has also been described 75.

Several medicinal plants and naturally occurring phytochemicals, such as curcumin, genistein, lectins, indoles, glucans, capsaicin, phytosterols, resveratrol, saponins, tannins, terpenoids, quercetin, polysaccharides, epigallocatechin-3-gallate, flavonoids, isoflavonoids, alkaloids and peptides, colchicine, fatty acids, sesquiterpenes, andrographolide, and labdane diterpenes, have shown to possess immunomodulating properties 74, 76. *Echinacea species*, which are widely used in the U.S. for their immunostimulant properties, affect both innate and adaptive immunity. It is known for its antiviral, anti-inflammatory, and antimicrobial effects 74. It also influences the immune system by modulating the gut microbiota 75. The immunomodulatory effects of alkylamines and polysaccharides are achieved through enhanced macrophage and lymphocyte activation. Recently, Ligustrum vicaryi L. fruit polysaccharide (LVFP), has been used as an immunomodulator. In an immunocompromised mouse study, LVFP administration increased the spleen and thymus indices. It also enhances neutrophil phagocytic activity, accelerates B and T-lymphocyte activation, and elevates the serum levels of Interleukin-10 (IL-10) and tumor necrosis factor alpha (TNF-α). These findings indicate that LVFP stimulates both innate and adaptive immunity and possesses anti-inflammatory properties 77. The health benefits of curcumin (present in *Curcuma longa L*.), including its immunomodulatory properties, are accepted worldwide 77-79. Immunomodulation is achieved by activating T-cells and other immune cells 80. A recent study conducted on the *Huh-7* cell line indicated that EGYVIR, a mixture of black pepper and curcumin extract, has antiviral activity against SARS-CoV-2. The virus is inactivated by suppressing the NF-kβ pathway, which reduces the release of Interleukin-6 (IL-6) and TNFα 80, 81 The role of turmeric in enhancing the immune system, particularly in respiratory diseases such as COVID-19, has been explored 79. A study on nanoparticle-mediated delivery of curcumin revealed that curcumin stimulated cell-mediated and humoral immunity in mice 77. Although plant-based immunomodulators have variable effects, they have immense scope due to their low toxicity and better efficacy when used in combination with other herbal or chemical immunomodulators (Table 7). Drug formulations, through the encapsulation of potential compounds in nanoparticles and their surface modification or capping of nanomaterials, serve as potent immunomodulatory nanotheranostics. Plant-derived immunomodulators serve as nanocarrier-based nanosystems and have been proven to be effective against autoimmune diseases, tumors, inflammation, immune cell function regulation, cytokine neutralization, enzyme-like activity, etc 82. Various formulations and alterations in administration methods, along with advancements in nanotechnology-based delivery systems, have addressed critical pharmaceutical challenges associated with the pharmacokinetics of curcumin, as listed in Table 8. These efforts aim to enhance its therapeutic efficacy, offering renewed optimism for the clinical application of this natural compound 83.

### Natural product-based nanotheranostics for inflammation

Inflammation is a complex immune response triggered by physical factors, leading to symptoms such as fever and fatigue. It involves the production of proinflammatory cytokines and chemokines, as well as reduced antioxidant defenses. Lipid intermediates from arachidonate metabolism also contribute to inflammation-related diseases such as cancer and cardiovascular issues. Inflammation is linked to various conditions, including cancer, obesity, neurodegenerative diseases, diabetes, and demyelination. Natural compounds with anti-inflammatory properties show promise for preventing or treating these conditions. Resveratrol is a well-studied polyphenolic compound with pleiotropic effects that is commonly found in grape skin, peanuts, legumes, and berries. It stimulates natural defense mechanisms in plants, along with beneficial effects in animals and humans, such as playing important roles in neuroprotection, anticancer activity, improving insulin sensitivity, increasing longevity, and exhibiting anti-inflammatory properties 84-88. Quercetin, a well-studied polyphenol, and flavonoid are found in onion, broccoli, herbs, wine, fruits, and tea. Quercetin plays a crucial role in preventing inflammation through the inhibition of inflammatory mediators and the suppression of lipoxygenase enzyme activity. Quercetin reduces the synthesis and secretion of inflammatory mediators and histamine, mainly by alleviating the cell membranes of mast cells 89, 90. Isoniazid and rifampicin are drugs that are routinely used to treat tuberculosis (TB); however, their use can cause severe liver damage, potentially leading to liver failure. Prophylactic quercetin treatment reduced oxidative stress caused by anti-TB drugs by enhancing NRF-2 activation, which in turn lessened the severity of liver inflammation and necrosis 91. *Opuntia ficus-indica,* commonly known as *Nopal cactus*, is used in traditional medicine in sub-Saharan regions and is sourced from various aerial parts, including cladodes, fruits, and flowers, and has antioxidant, flavonoid, neuroprotective, hepatoprotective, and anticancer activities 33, 92. In addition, extracts derived from *Nopal cactus* exhibit neuroprotective activity by inhibiting nitric oxide production and increasing antioxidant activity in microglial BV-2 cells 93. The aqueous extracts from tomatoes reduced inflammation in macrophages. In parallel, the expression of interleukins and chemokines in human peripheral blood leukocytes (PBLs) is altered, suggesting that aqueous tomato extract blunts inflammation and enhances immune regulation 94. An herbal medicine, KIOM-MA, which is used to treat atopic dermatitis and asthma, also suppressed proinflammatory mediators in lipopolysaccharide-stimulated RAW 264.7 cells by attenuating the LPS-induced increases in nitric oxide synthase, COX-2, nitric oxide, and PGE2. PGE_2_ were blocked by the administration of KIOM-MA *in vitro* 95.* Potentilla erecta* (PE) belongs to the *Rosaceae* family and is traditionally used to treat diarrhea, hemostasis, and hemorrhoids. PE inhibited UVB-induced upregulation of COX-2 and dose-dependently reduced the level of PGE_2_. These findings suggest that PE fractions containing high amounts of agrimoniin play an anti-inflammatory role *in vitro* and *in vivo* 96. A derivative of 17-hydroxy-jolkinolide B (HJB-1) exhibited an anti-inflammatory effect against LPS-induced acute respiratory distress syndrome (ARDS) *in vitro*. HJB-1 markedly reduced LPS-induced pulmonary histological changes, lung edema, inflammatory cell infiltration, as well as cytokine, NF-kB, and MAPK activation 97. Koumine is an alkaloid extracted from *Gelsemium elegans* that blocks LPS-induced ROS production, p53 activation, and mitochondrial dysfunction in RAW 264.7 macrophages, suggesting that koumine potentially has a protective effect against LPS-induced damage 98. The bark of *A. occidentale* L. demonstrates various biological activities, including antioxidant, antimicrobial, and anti-inflammatory effects. The ethyl acetate extract (EtOAc) of *A. occidentale* was found to reduce edema and lower levels of IL-1β and TNF-α, indicating that EtOAc plays a critical role in modulating the inflammatory response in a preclinical model 99-101. NPs and nano-emulsions are employed to target inflammation by recognizing molecules expressed on the surface of inflammatory cytokines or endothelial cells, taking advantage of increased vascular permeability, or using biomimicry 102. These strategies offer promising approaches for treating inflammatory diseases. The widespread use of natural compounds for preventing and treating various diseases is attributed to their antioxidative and anti-inflammatory properties. As promising natural compounds, plant-derived exosome-like nanoparticles (PELNs) are derived from the multivesicular bodies of various edible plants, including vegetables, foods, and fruits. These nanoparticles can effectively restore the balance between proinflammatory and anti-inflammatory effects in a range of diseases, including colitis, cancer, and inflammation-related metabolic disorders 103. Recent scientific interest has focused on the use of extracellular vesicles (EVs) derived from natural compounds as potential treatments for inflammatory diseases. These nanometer sized EVs, which are isolated from plants such as grapefruit and *Dendropanax mellifera*, are efficiently taken up by host organs and can influence both physiological and pathological processes 104. Additionally, nanoparticles made from albumin and cerium oxide, synthesized through biomineralization, exhibit enzymatic-like properties and can scavenge ROS. These nanoparticles reprogram macrophages from a proinflammatory state to an anti-inflammatory state. In the collagen-induced arthritis (CIA) mouse model, the nanoparticles accumulated in inflamed joints and demonstrated therapeutic efficacy comparable to that of established rheumatoid arthritis treatments 105. Table 9 summarizes some studies pertaining to plant-based nanotheranostics for inflammatory disorders.

### Natural product-based nanotheranostics for hepatoprotective properties

Natural bioactive components sourced from plant secondary metabolites are increasingly recognized as valuable alternatives for preventing and alleviating hepatotoxic effects and their chronic complications. They possess antifibrotic, antioxidant and antihepatotoxic properties. This review delves into the mechanisms of action of promising plant-derived products for treating patients with various liver disease 106. The incorporation of nanotheranostic agents such as nanoparticles and carbon-based nanoderivatives further improves the theranostic efficiency of beneficial hepatoprotective regimens. The hepatoprotective activity of silver NPs has been demonstrated using a plant extract of *Rhizophora apiculata* as a reducing agent to prepare NPs. This study incorporated a mouse model induced with carbon tetrachloride, and the activity of* R. apiculata* with silver NPs was studied. As a result, the nanoparticles exhibited hepatoprotective effects that were derived from mangrove ecosystem plant extracts 107. Another report by *Li et al*. described a silibinin-loaded hyaluronic acid (HA) micelle. This micelle binds specifically to CD44 receptors, which are abundant on the membrane of activated hepatic stellate cells (aHSCs). These micelles exhibited exceptional targeting capabilities toward fibrotic liver tissues and selectively bound further, eliminating aHSCs 108. Consequently, this approach has demonstrated a highly effective anti-hepatic fibrosis effect. Various natural products for combined components of hepatoprotective disorders are tabulated below in Table 10.

## Nonalcoholic fatty liver disease (NAFLD)

Nonalcoholic fatty liver disease (NAFLD) is a chronic liver condition with limited treatment options. Clinically, it encompasses a wide range of hepatic anomalies, that includes nonalcoholic steatohepatitis (NASH), simple steatosis, fibrosis, and cirrhosis 109. The accumulation of intolerant levels of fats in the liver (other than alcohol abuse) is characteristic of NAFLD 110. NAFLD is reportedly associated with other metabolic disorders, such as type II diabetes, hyperlipidemia, obesity, CVD, and chronic kidney disease (CKD) 111-113. In the recent past, the incidence of NAFLD has increased drastically due to dietary changes and urbanized lifestyles 110, 114. The results of several studies suggest that natural bioactive components of herbs and the diet can be studied for the prevention and treatment of NAFLD. The Qushi Huayu Fang (QHF) mixture of five herbal medicines, *Polygonum cuspidatum*, *Curcuma longa*,* Artemisia capillaris*, *Gardenia jasminoides*, and *Hypericum japonicum*, is traditionally used in China for the treatment of NAFLD 115. In a study on NAFLD rats, QHF administration decreased the deposition of fat in liver and recovered liver pathology through reduced fat degeneration, hepatocyte ballooning, and inflammation 112. Several other mechanisms for the anti-NAFLD activity of QHF are also discussed, including the upregulation of peroxisomal proliferator-activated receptor α (PPARα), regulation of adipocytokine release, the suppression of insulin signaling, the downregulation of Sterol Regulatory Element Binding Protein-1c (SREBP-1c) levels, the suppression of systemic and macrophage inflammation, and the modulation of the gut microbiome 115 In China, for several years, Fufang-Zhenzhu-Tiao Zhi Capsule (FTZ), a cocktail of eight Chinese medical herbs, improved NAFLD in mice by altering the gut microbiota. An increased Bacteroidetes ratio and a decreased Firmicutes/Bacteroidetes ratio are correlated with reduced lipogenic gene expression, including genes such as *SCD1*, *FAS*, *CD36*, and *C/EBP-α* 116. Phloroglucinol (PHG), a phenolic compound found in some plants, is used to treat gastric disorders. A study on HepG2 cells exposed to hydrogen peroxide or palmitic acid revealed that PHG has strong antioxidant properties that are comparable to those of α-lipoic acid (ALA) and N-acetylcysteine (NAC). PHG also reduces inflammation and apoptosis in treated cells 117. Another study demonstrated the therapeutic effect of vine tea polyphenol (VTP), a Chinese herb derived from *Ampelopsis grossedentata*, in WD-induced NAFLD mice (C57BL/6 N). These results suggest that dihydromyricetin (a bioactive component of VTP) reverses the effects of WD by decreasing the serum triglyceride and cholesterol levels, decreasing the accumulation of lipids in the liver, and modulating the gut microbiota 118. Recent advancements in the use of polymeric nanoparticles such as nanocurcumin have led to the investigation of diverse techniques to address hepatic disorders. For example, the use of polylactic-co-glycolic acid (PLGA) nanoparticles has resulted in a 22-fold increase in curcumin bioavailability. Curcumin has the potential to enhance the onset and progression of NAFLD by effectively inhibiting inflammation and oxidative stress 119.

## Alcoholic Liver Disease (ALD)

Chronic alcohol consumption causes liver damage and is considered as one of the most common causes of chronic liver disease 120. According to the 2018 WHO report, 3 million deaths are reported every year due to alcohol abuse, accounting for 5.3% of the total deaths worldwide. ALD causes several changes in the liver, from simple steatosis to cirrhosis and acute alcoholic hepatitis 121. The global burden of ALD is increasing, yet treatment options remain limited. The clinical presentation of ALD varies among individuals and changes over time. Herbal products, with their multiple targets and fewer side effects, offer potential for preventing cirrhosis and improving patient life expectancy 122, 123. *Silybum marianum* derived flavonoid, Silymarin, is widely used in the treatment of liver disease. A recent meta-analysis revealed that it acts as an antioxidant, reduce ROS production and lipid peroxidation and thereby protects cells.

This study also revealed that oral silymarin lowers serum alanine aminotransferase (ALT) and aspartate Aminotransferase (AST) levels, although the reduction was not clinically significant, warranting further research. Its anti-inflammatory effects are achieved by inhibiting NF-κB, which reduces the levels of inflammatory cytokines in liver cells 124. In an ethanol-induced mouse model, Pueraria lobata and Silybum marianum showed greater efficacy in reducing steatosis and hepatic inflammation when used together than when used alone. This effect was linked to enhanced LKB1/AMPK/ACC signaling and reduced LPS-triggered TLR4-mediated NF-κB signaling 125. Garlic (*Allium sativum L.* [Amaryllidaceae]) is traditionally used to treat liver disease 126. Garlic polysaccharide (GP) has a significant hepatoprotective effect on ALDmodel mice. GP treatment reduced oxidative stress and lipid peroxidation and modified three pathways, namely, TNF-α, TGF-β1 and decorin pathways, to prevent hepatic stellate cell (HSC) activation and reduce the production of the extracellular matrix (ECM). Further modulation of the gut microbiota by GP treatment has also been reported 127. Glycyrrhizin, obtained from the roots of *Glycyrrhiza glabra* (licorice), exhibits potent hepatoprotective properties. In one study, its impact on alcohol consumption was evaluated through a randomized, placebo-controlled, double-blind, trial involving 12 healthy participants (six males and six females). The results suggest that, compared with alcohol intake without glycyrrhizin, the intake of alcohol with glycyrrhizin improves liver function 128. In another study, mice were given alcohol with or without licorice. The negative effects of alcohol consumption were reversed in the mice treated with licorice, a result attributed to its antioxidant and anti-inflammatory properties 129. Conventional diagnostics and drug treatments often lack precision and effectiveness. Nanomedicine, a promising field in medical nanotechnology, offers improved imaging, enhanced tissue penetration, and targeted drug delivery for alcoholic liver disease. It also combines diagnosis and therapy, functioning as a nanotheranostic 130. The hepatoprotective effect of ginger against certain types of toxicity has been studied extensively. Shogaols, which are dehydrated gingerol analogs, are a major focus of research pertaining to anti-inflammatory effects in the liver 131. Quercetin is also used in nanotheranostics, as demonstrated by a study that developed a more biologically available liposomal formulation to assess its effects on liver damage in male rats. Researchers investigated the effects of nanoliposome-delivered quercetin on liver damage induced by amoxicillin/clavulanate, focusing on its impact on the NF-κB/SIRT1/Nrf2 signaling pathway and microbiota modulation. The study found reduced hepatic damage, as indicated by improved serum liver enzymes, enhanced antioxidant status, and changes in microbiota 132.

## Viral hepatitis

Viral hepatitis is a major health issue representing a systemic infection that affects mainly the hepatic system 133-135. Five classes of viruses have been identified and named hepatitis A, B, C, D, and E. All five strains affect liver function but differ in their structure, transmission mode, lethality, preventive technique, and geographical spread. At present, no vaccines are available for all classes of viral infection. Owing to the differences mentioned above, the vaccine developed for one type of virus cannot be 100% effective against other classes. Additionally, due to the adverse effects and high cost of the vaccine, attention to alternative treatment strategies, such as traditional herbs, has been explored to identify a cure for viral infection. Glycyrrhizic acid (GA), a bioactive component of liquorice, has been shown to improve hepatic inflammation (murine hepatitis virus (MHV)-A59-induced model) by modulating the HMGB1-TLR4 signaling pathway. The protective effect of GA treatment was due to reduced IL-17 and IL-22 levels 136. A study highlighted the importance of several Indian medicinal plants, including Phalatrikadi Kwath, Triphala, Vasa (*Adhatoda vasica*), Giloy (*Tinospora cordifolia*), Neem (*Azadirachta indica*), Kiratatikta (*Swertia chirayita*), Katuki (*Picrorhiza kurroa*), Kalmegh (*Andrographis paniculata*), Musta and Nagaramustaka (*Cyperus rotundus* and *Cyperus eleusinoides*), and Bhumyamalaki (*Phyllanthus niruri*), for their hepatoprotective role against hepatitis infection. The significant advantages of nanoparticle-based drug delivery systems have paved the way for developing strategies targeting the hepatitis B virus (HBV) 137. There has been a notable increase in various nanoparticle formulations for anti-HBV therapy, integrating features from different delivery systems used in anti-HBV approaches such as vaccines, gene therapy, and nucleoside drugs 9. Various nanotheranostic agents, such as lipid molecules and polymeric micelles, have been effectively employed for developing drug delivery systems for anti-HBV-based gene therapy, and RNA interference (RNAi) therapeutics have been used for antiviral treatment of plant-based products 138. From this perspective, herbal polymer-based nanomedicines can be easily tailored for optimal physicochemical attributes and functions, offering significant advantages in treating viral hepatitis. Compared with conventional therapeutic agents, they contribute to improved therapeutic outcomes and mitigate adverse drug effects 139. Various studies have reported that plant-derived nanovesicles (PNVs), which are natural disease therapeutics, are eco-friendly, have low toxicity, high yield, simplicity, safety, etc., and have a low risk of *in vivo* immunogenicity 140. There are certain examples listed in the literature in Table 11 consisting of various plant-based products for nanotheranostics in viral hepatitis.

## Summary

The literature suggests that natural plant products exhibit significant biological and pharmacological activities. Although plant-based medicines have been used for centuries, their effectiveness is often limited by poor bioavailability, low stability, and nonspecific targeting. Recent advancements in nanotechnology have introduced the concept of "nanotheranostics," which combines therapeutic and diagnostic functions within a single nanostructure. By incorporating plant-based compounds into nanoparticles, researchers have developed innovative nanotheranostic agents. These agents significantly enhance drug delivery, targeted therapy, and disease diagnosis. These nanotheranostic agents work by encapsulating plant-derived active ingredients within nanoparticles, which are engineered to target specific cells or tissues. The nanoparticles can be designed to release the drug in a controlled manner, ensuring that the therapeutic compounds reach the diseased site with minimal side effects on healthy tissues. Additionally, these nanostructures can be equipped with imaging agents that allow real-time monitoring of drug delivery and disease progression through various imaging techniques, such as MRI, PET, or fluorescence imaging. The integration of plant-based medicines with nanotechnology offers a promising approach for more effective and personalized treatments. Nanotheranostic not only improve the therapeutic efficacy of plant-derived drugs but also provide tools for early and accurate disease diagnosis, making them a powerful asset in modern medicine. Important benefits of nanotheranostic agents are also illustrated in Fig. 3.

## Future Directions

The integration of plant-derived natural products with nanotechnology holds immense potential for advancing healthcare. Future research should focus on optimizing the synthesis and functionalization of nanotheranostic agents to enhance their biocompatibility, targeting efficiency, and therapeutic efficacy. The development of multifunctional nanoparticles that can simultaneously diagnose, deliver drugs, and monitor treatment response in real-time will revolutionize personalized medicine. Additionally, exploring the use of plant-based exosomes and other nanovesicles as delivery systems could open new avenues for non-invasive diagnostics and targeted therapies. Collaborative efforts between multidisciplinary teams, including chemists, biologists, and clinicians, will be crucial in translating these innovations from the laboratory to clinical practice. By harnessing the unique properties of natural products and advanced nanomaterials, we can pave the way for more effective, safe, and personalized treatment strategies for a wide range of diseases.

## Figures and Tables

**Table 1:** Plant products that regulate blood sugar levels and control diabetes

Plant Product	Importance in Health	Mechanism of Action
Cannabinoid type 1 receptor (CB1R)	Stimulates endoplasmic stress, contributing to insulin resistance and increased gluconeogenesis.	High fat diet (HFD)-induced increased blood glucose and insulin level and downregulation of HFD-induced ER stress, gluconeogenic gene, expression of CB1R, and ceramide synthesis 20
Onion	Exhibits anti-inflammatory, antioxidant, and antidiabetic values due to flavonoids compounds such as quercetin and organosulfur compounds	The Amador rearrangement compound suppressed the absorption of carbohydrates by blocking the intestinal sucrose 141
Myricitrin	Antioxidant, anti-diabetic, and antiapoptotic effects in mice and myotube cells	Myricitrin-loaded SLNs enhanced β-cell function, improving hyperglycemia through increased insulin levels 142
Aqueous bark extract of *Saraca asoca*	Testing wound healing efficacy by studying the expression of inflammatory factors like cytokines and pro-inflammatory markers in diabetic mice	Green-synthesized silver nanoparticles effectively killed multidrug-resistant bacteria, showing promise as a safe topical treatment for diabetic wounds 143
Oleanolic acid and Polygalacturonic acid	Anti-diabetic properties	The nanocomplex micelles promoted IRS-1/PI3K/AKT signaling pathway via inhibition of PTP1B enzyme 144
Stevia Glycosides	Anti-diabetic nanoformulation	Oral bioavailability and target specificity; Nanotheranostic agent as Stevioside-assembled Polyethylene glycol nanoparticles 145
Mulberry leaf and Pueraria Lobata extracts	Hypoglycemic effects	Slow drug release and Intestinal permeability; Nanotheranostic agent as Mulberry leaf and Pueraria Lobata extracts loaded selenium-layered nanoparticles 146
Emodin	Diabetic management	Attenuate diabetes-provoked neuropathic pain via suppressing purin 2X3 (P2X3) receptor expression; Nanotheranostic agent as Emodin-loaded poly-PEGMA-DMAEMA-MAM nanoparticles 147

**Table 2:** Important role of plants produced in neurodegenerative disorders

Plant Product	Neurodegenerative Disease	Mechanism of Action
*Lippia citriodora* extract and verbascoside	PD, HD and AD	Regulating genes responsible for maintaining calcium homeostasis and energy production, enhancing the expression of serotonin, brain-derived neurotrophic factor (BDNF), and dopamine 30
Auraptene (found in citrus fruit)	PD	Inhibiting mitochondrial respiration and reducing ROS generation 32
*Chionanthus retusus* extract	PD, HD, AD, MS and ALS	Blocking LPS-induced NO production and neuroprotection against cellular toxicity induced by glutamate 148
*Centella asiatica* extract	AD	Attenuating cognitive decline, inhibiting morphological aberration in the hippocampus, decreasing the level of glycogen synthase kinase-3 beta, and increasing protein phosphatase 2 level 149
Thymol (found in various plants)	PD	Attenuating oxidative stress, neuronal loss, and inflammation in a rat model of Parkinson's disease induced by rotenone 150

**Table 3:** Plant product-derived nanotheranostics for the control of neurodegenerative disorders

Plant Product	Importance in Health	Mechanism of Action	Nanotheranostic agent
Chitosan-gelatin-green tea extract	PD	Generation of reactive oxygen species, increased expression of tyrosine hydroxylase (TH) enzyme, and decreased expression of α-syn protein.	Chitosan-gelatin-green tea extract nanoparticles 151, 152
*Ephedra sinica Stapf* extract	Neuroinflammation	Anti-neuroinflammatory properties	Gold nanoparticle 153
Ferulic acid (FA)	AD	Exhibits antioxidant, anticancer, cardioprotective, and neuroprotective properties.	Chitosan-coated solid lipid nanoparticles 154
Berberine	AD	Suppressed AChEI activity and mitigated memory loss.	Multi walled Carbon Nanotubes coated with phospholipid and polysorbate 155
Retinoic acid	PD	Inhibit α-syn aggregation *in vitro*	Chitosan nanoparticles and nanomicelle 154

**Table 4:** Plant product-derived nanotheranostics for the control of malarial infections

Plant Product	Importance in Health	Mechanism of Action	Nanotheranostic agent
*Plectranthus amboinicus* leaf extract	Antimalarial effect	Disruption of epithelial layer and vacuolization of cells of larvae	Zinc oxide nanoparticles 156
*Cymbopogon citratus* leaf extract	Antimalarial effect	Control of Anopheles and Aedes larval populations	Gold NPs 157
Mimosa pudica Gaertn leaf extract	Antiparasitic activity	Control larvae of plasmodium, *Anopheles subpictus* Grassi, and *Rhipicephalus*	Silver NP 158
Curcuminoids	Antiparasitic activity against* Plasmodium berghei*	Cured infected mice models and showed recrudescence	Liposomes 159
Artemisinin	Parasitemia inhibition	Intravenous administration of artemisinin	Artemisinin nanoparticles 160

**Table 5:** Plant products demonstrate effectiveness in cancer models

Plant Products	Cancer Type	Mechanism of Action
Coffee	Various types of cancer	Induction of cell death, antioxidant and anti-inflammatory effects, DNA damage prevention, antiproliferative activity, angiogenesis inhibition, and suppression of matrix metalloproteases 54
Carotenoids (β-carotene, curcumin, lutein, phytoene, crocin, crocetin, lycopene, β-cryptoxanthin, and astaxanthin)	Breast, ovarian, cervical, and colorectal cancer	Apoptosis induction, gap junction modulation, cell cycle regulation, antiproliferative action, growth factor signaling control, antioxidant response activation, and modulation of differentiation-related proteins 58, 59
Resveratrol	Skin, breast, colon, and liver cancer	Modulating apoptosis through the BAX gene, inhibiting metastasis by repressing the MALAT1 governed EMT 62
EGCG	Prostate, brain, bladder, cervical cancer	Inhibits cell proliferation, extends rat lifespan, upregulates p21^Waf1/Cip1^, downregulates CDC25A, and induces cancer cell apoptosis via epigenetic regulation of genes like hTERT or by increasing ROS levels 161

**Table 6:** Plant product-derived nanotheranostics for the control of cancer

Plant Product	Importance in Health	Mechanism of Action	Nanotheranostic agent
Noscapine	Prostate cancer	Imaging and drug delivery	Iron oxide NPs 162
Resveratrol	Breast cancer	Cytotoxic activity	Polymeric NPs 163
Gelatin	Tongue squamous cell carcinoma cells	Cisplastin loaded CNTs as drug delivery agent	Carbon nanotubes 164
Olive-leaf extracts	Breast, Colorectal, Hepatocellular cancer	Anticancer effect	Multiwalled carbon nanotubes 164
*Salvia spinosa* extract	Pancreatic cancer	Cytotoxic activity	Graphene oxide 165

**Table 7:** Immunomodulatory role of plant products

Plant-Derived Product	Source	Immunomodulatory Effects
Resveratrol	Grape skin, peanuts, legumes, berries	Stimulates natural defense mechanisms, anti-inflammatory, neuroprotective, anticancer, improves insulin sensitivity, increases longevity 62
Quercetin	Onion, broccoli, herbs, wine, fruits, tea	Inhibits inflammatory mediators, suppresses lipoxygenase enzyme activity, alleviates mast cell membranes, attenuates drug-induced oxidative stress, inhibits mast cell activation-induced release of cytokines 166
Opuntia ficus-indica	Nopal cactus (cladodes, fruits, flowers)	Antioxidant, neuroprotective, hepatoprotective, anticancer, inhibits nitric oxide production, increases antioxidant activity 93
Aqueous extract from tomatoes	Tomatoes	Reduces inflammation in macrophages, alters expression of interleukins and chemokines, improves immune regulation 94
KIOM-MA	Herbal medicine	Inhibitory effect on pro-inflammatory mediators in LPS-stimulated cells, used to treat atopic dermatitis and asthma 95
*Potentilla erecta* (PE)	Rosaceae family	Inhibits ultraviolet-B (UVB)-induced upregulation of cyclooxygenase-2 (COX-2), reduces level of Prostaglandin E2 (PGE2), exhibits anti-inflammatory role both *in vivo* and *in vitro* 96
17-hydroxy-jolkinolide B derivative (HJB-1)	Unknown source	Attenuates LPS-induced pulmonary histological alteration, lung edema, inflammatory cells infiltration, cytokines, NF-kB, and MAPK activation 97
Koumine	Gelsemium elegans	Blocks ROS production induced by LPS, p53 activation, and mitochondrial dysfunction 98
Ethyl acetate phase (EtOAc) of *A. occidentale*	*A. occidentale L* bark	Reduces edema and levels of IL-1β and TNF-α 167

**Table 8:** Plant product-derived nanotheranostics for immunomodulatory effects

Plant Product	Importance in Health	Mechanism of Action	Nanotheranostic agent	Reference
Curcumin	Immunomodulatory effect with reduced replication of Human Immunodeficiency Virus (HIV)	Antiretroviral agents, inhibiting expression of pro-inflammatory mediators induced by HIV-1 infection	Silver nanoparticles	83
Curcumin	Modulating vascular deposition of circulating tumor cells	Prevent metastasis, modulate vascular inflammation	Lipid-polymer nanoparticles	168
*Hypoxis hemerocallidea-* Hypoxoside	Immunomodulatory effects in macrophages and Natural killer cells (NK)	Lower pro-inflammatory cytokine levels in macrophages. Reduce expression of cytokines in NK cells	Gold NPs	169

**Table 9:** Plant product-derived nanotheranostics for inflammatory disorders

Plant Product	Importance in Health	Mechanism of Action	Nanotheranostic agent
*Anoectochilus elatus* Leaf Extract	Anti-inflammatory	inhibition of pro-inflammatory cytokines and suppression of NF-κB signaling pathways.	Silver nanoparticles 105
Ginger	Ulcerative colitis	reduced expression of CD98	Ginger-derived lipid nanoparticles 170
Puerarin, a flavonoid from* Pueraria lobata*	Anti-inflammatory	M1 macrophages polarization to M2	Exosome-like nanoparticles 171
Blueberry	Vascular system	Regulate the expression of inflammatory genes in response to TNF-α	Exosome-like nanoparticles 172
*Panax ginseng* leaves	Anti-inflammatory	Blockade of NF-κB activation in macrophages	Gold NPs 173

**Table 10:** Plant product-derived nanotheranostics for hepatoprotective disorders

Plant Product	Importance in Health	Mechanism of Action	Nanotheranostic agent
Curcumin	Hepatic cancer	Drug delivery system for hepatoma cells	Lactosylated curdlan-triornithine nanocarriers 174
Blueberry	NAFLD	Reduce oxidative stress, reactive oxygen species, prevented cell apoptosis in hepatic cells	blueberry-derived exosomes-like nanoparticles 175
*Celastrol*	NAFLD	Hepatoprotective activity	Albumin-based nanoparticles 176
Chitosan and Silymarin	NAFLD	Protective effect against liver steatosis	Polymer hybrid nanoparticles 177
Nicotinamide and ascorbic acid nanoparticles	NAFLD	Decreased oxidative and nitrosative stresses	Chitosan nanoparticles 178
Glycyrrhizin	Hepatocyte drug delivery	Decline of the tissue damage	Chitosan nanoparticles 179
Ginger	ALD	Inhibited reactive oxygen species production	Ginger extracts nanoparticles 180
Quercetin	Liver cancer	Inactivation of signaling pathways causing cancer	Gold-quercetin nanoparticles 180
Quercetin	ALD	activates autophagy and restore lysozyme function by combating exosomes release	Quercetin exosomes 181
Silymarin and quercetin	NAFLD	hepatoprotective activity	Miniaturized scaffold 182
Silymarin	ALD	Inversion of biochemical parameters and histopathological alterations	Silymarin Phytosome nanoparticles 183
*Glycyrrhiza glabra* (Licorice) root extract	Liver disease	Anti-Inflammatory effect in liver diseases	Nanoparticles 184, 185

**Table 11:** Plant product-derived nanotheranostics for viral hepatitis

Plant Product	Importance in Health	Mechanism of Action	Nanotheranostic agent
Mannose	Hepatitis B vaccine delivery	Hepatitis B surface antigen loaded inside MNP strengthened humoral and cellular immune responses	Mannose-modified poly D, L-lactide-co-glycolic acid (PLGA) nanoparticles (MNP) 186
Ferritin	Chronic hepatitis B	Ferritin NPs delivering preS1 as vaccine candidate including SIGNR1+ dendritic cells	Ferritin nanoparticle 187
Stearic acid and Chitosan	Hepatitis B virus	Inhibition of HBeAg expression by complex micelle and 10-23 DNAzyme	Complex Micelle 188
Chitosan	Hepatitis B Virus	Inhibitory effects on antigen expression and DNA replication	Chitosan micelles containing Lamivudine stearate 189
